# Advancing Personalized Medicine in Gestational Diabetes Mellitus Management Through Gut Microbiota Insights

**DOI:** 10.3390/ijms27146424

**Published:** 2026-07-20

**Authors:** Abdoulaye Diane, Razik Bin Abdul Mu-U-Min, Heba Hussain Al-Siddiqi

**Affiliations:** 1 Diabetes Research Center, Qatar Biomedical Research Institute (QBRI), Hamad Bin Khalifa University (HBKU), Qatar Foundation (QF), Doha P.O. Box 34110, Qatar; 2 College of Health & Life Sciences, Hamad Bin Khalifa University (HBKU), Qatar Foundation (QF), Doha P.O. Box 34110, Qatar

**Keywords:** pregnancy, gut microbiota, dysbiosis, gestational diabetes mellitus, pathogenesis, microbiomics, biomarkers, precision medicine

## Abstract

Gestational diabetes mellitus (GDM) is a metabolic disorder that is characterized by hyperglycemia developed or first diagnosed during pregnancy, affecting up to 15% of pregnant women worldwide. Although risk factors for GDM such as a family history of diabetes, obesity, and advanced maternal age are well recognized, the underlying pathophysiological mechanisms are complex and remain incompletely understood. Multiple studies indicate that GDM arises from a combination of peripheral insulin resistance and pancreatic β-cell dysfunction, in which insulin secretion fails to compensate for the progressive increase in insulin demand during pregnancy. In recent years, growing attention has been focused on the gut microbiota and its potential influence on the development and progression of many metabolic diseases, including GDM. Studies have shown that women diagnosed with GDM exhibited altered gut microbial composition and reduced microbial diversity, termed as “dysbiosis”, suggesting that gut microbiota dysbiosis may contribute to the pathogenesis of GDM. However, most evidence in humans is associative rather than causal, and findings vary across populations due to differences in study cohort characteristics (GDM diagnostic criteria, ethnicity, lifestyle, dietary habits, pregnancy body mass index, gestational age) and sequencing methods. Current treatment guidelines for the management of GDM are universally standardized, despite the known heterogeneity of the disease etiology. Given the inter-individual differences in gut microbial composition in women with GDM, the gut microbiota has been proposed as a promising source for rapid and specific biomarkers to enable early diagnosis, targeted prevention, and personalized management of GDM. In this narrative article, we first summarize current insights into the pathophysiological mechanisms linking gut microbiota to the development of GDM. Second, we emphasize the importance of gut microbiota analysis (or microbiomics) within multi-omics frameworks for the early detection, prevention and personalized management of GDM.

## 1. Introduction

Gestational diabetes mellitus (GDM) represents a subcategory of diabetes mellitus characterized by hyperglycemia that typically develops or is diagnosed during the second or third trimester of pregnancy [1,2]. GDM is one of the most common endocrine disorders in pregnant women [3], affecting approximately up to 15% of pregnancies worldwide [4,5]. It is associated with significant short- and long-term health risks for both the mother (e.g., pre-eclampsia, pre-term delivery, metabolic syndrome and cardiovascular diseases) and the fetus (e.g., macrosomia and hypoglycemia) [6], making it an emerging health concern. GDM is a transient condition that manifests during pregnancy, with normoglycemia restored shortly after delivery. However, approximately up to 50% of women with GDM are at risk of developing type 2 diabetes (T2DM) within 5 years postpartum (cumulative incidence) [7]. The incidence of T2DM among Danish women with previous GDM had more than doubled over a 10-year period (from 18.3% in the old cohort at the 1990 follow-up to 40.9% in the new cohort at the 2000 follow-up) due to a substantial increase in BMI in women with GDM [8]. In a follow-up cohort study from 1960 to 2009, Bellamy et al. reported that women who have had GDM have at least a seven-fold increased risk of developing T2DM in the future compared with normoglycemic pregnant women [8,9]. Moreover, the offspring of women with GDM have a higher risk of developing metabolic syndrome and cardiovascular disease in the future when compared to those born from mothers without GDM [10]. Thus, the effective control of blood glucose levels during GDM is crucial for reducing serious perinatal morbidity [11]. Based on Carpenter and Coustan diagnostic criteria, the International Association of Diabetes and Pregnancy Study Group (IADPSG) and the American Diabetes Association recommend that all pregnant women maintain fasting glucose levels below 92 mg/dL, 1 h postprandial glucose levels below 180 mg/dL or 2 h postprandial glucose levels below 153 mg/dL using the 75 g oral glucose tolerance test (OGTT). In comparison, the American College of Obstetricians and Gynecologists (ACOG), using a two-step screening approach at ≥24 weeks of gestation and a 50 g OGTT followed by a 100 g OGTT, recommends fasting glucose < 95 mg/dL and postprandial blood glucose < 180 mg/dL at 1 h, <155 mg/dL at 2 h and <140 mg/dL at 3 h as the cutoff values [12,13]. Although the exact etiology of GDM is not fully understood, evidence suggests that it is driven by a combination of genetic, epigenetic, and environmental factors [14,15,16]. The rising prevalence of GDM, along with other metabolic diseases such as obesity and T2DM, in genetically stable populations suggests that contemporary environmental factors, such as dietary habits, are likely to be major contributors to the disease etiology [17,18,19]. In recent years, increasing attention has been directed toward the gut microbiota as one of the environmental factors contributing to the development and progression of GDM [20,21]. Compared with healthy pregnant women, women diagnosed with GDM showed altered gut microbial composition, referred to as “dysbiosis” [2,22]. This suggests that GDM is associated with a specific gut microbiota signature that may play a role in its pathogenesis, as demonstrated by phenotype transfer in mice following fecal microbiota transplantation from pregnant women with GDM [23,24]. In this study, mice receiving transplants from women with GDM exhibited impaired glucose tolerance, which was reversed after receiving transplants from the same women following dietary intervention, highlighting how dietary interventions can effectively manage GDM, at least in part, by modifying the gut microbiota. Despite the known heterogeneity in the etiology of GDM, current treatment guidelines remain widely and universally standardized [25,26,27]. Standard care typically includes lifestyle and dietary counseling, regular blood glucose monitoring and, when necessary, pharmacological intervention with antidiabetic drugs such as metformin or insulin. However, approximately one-third of pregnant women fail to achieve glucose targets through lifestyle-based interventions alone [28], indicating a heterogeneity, as for the etiology, in response to standard GDM care. Therefore, the identification of biomarkers through multi-omics studies, including microbiomics, could offer a unique and specific tool for early detection and treatment of GDM. Unlike genetic factors, gut microbiota is easily modifiable by exogeneous factors including diet and physical activity, therefore receiving increasing attention as a promising rapid and specific target for personalized management strategies of GDM. Interestingly, previous reports used single omics (microbiomics) to address the gut microbiota–GDM axis [22,29,30]. For taxonomic biomarkers of GDM, Ferrocino et al. reported a trimester-specific gut microbiota signature in GDM and, more specifically, identified associations between specific bacteria taxa and metabolic parameters [22]. Furthermore, Crussell et al., using 16S rRNA gene sequencing, demonstrated that women diagnosed with GDM at late pregnancy (third trimester) exhibited aberrant gut microbiota composition that persisted until an average of 8.8 months after delivery [30]. Additionally, in a systematic review, Ma et al. reported a significant association between gut microbiota composition/diversity and GDM development [29]; however, integration with host genetics (a risk factor for GDM) is needed to validate these findings for potential clinical application. Therefore, omitting the integration of other omics (e.g., genomics, transcriptomics, proteomics, metabolomics) into microbiomics might limit the identification of clinically translatable biomarkers for precision medicine. In this narrative review, we first summarize current insights into the pathophysiological mechanisms linking gut microbiota to GDM and then highlight the importance of gut microbiota within multi-omics frameworks for the early detection, prevention and personalized management of GDM, which may offer significant benefits for both the mother and the fetus.

The methods used in this review are as follows: First, we carried out a comprehensive search of peer-reviewed original and review articles using the PubMed database and Google Scholar without a time window restriction using the following search term combinations: “gestational diabetes”, “diabetes mellitus”, “type 2 diabetes mellitus”, “gut microbiota”, “microbiomics”, “omics” and “precision medicine”. Secondly, the reference section of each selected article was explored for additional articles.

## 2. Gut Microbiota Diversity

**Figure 1:**
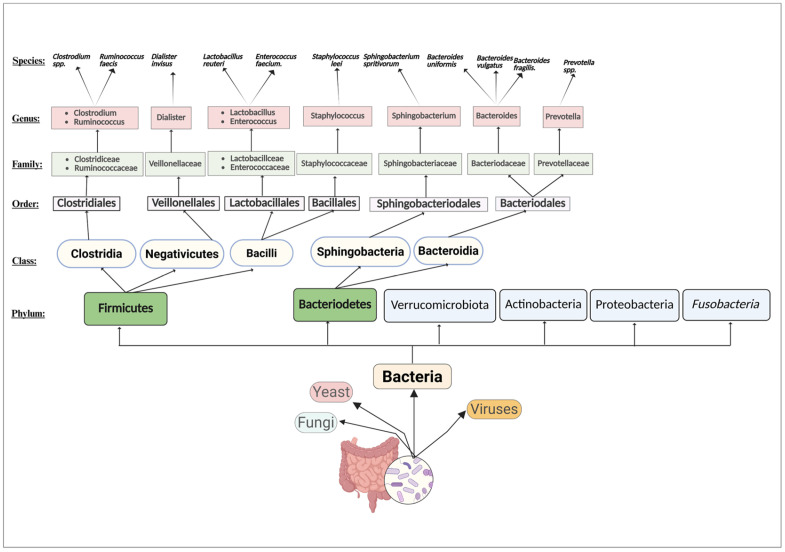
Taxonomy of the gut microbiota, with the main phyla dominating the gastrointestinal tract microbiota. The box shows examples of bacterial species belonging to Firmicutes and Bacteroidetes that represent ~90% of the gut microbiota. Created in BioRender. Al-Siddiqi, H. (2026) https://BioRender.com/ow7eumn (accessed on 16 April 2026).

The gut microbiota, known as a “virtual endocrine metabolic organ” or “the second genome” [31,32], colonizes the human gastrointestinal (GI) tract and is a complex ecosystem composed of several species of microorganisms, including yeast, viruses, fungi and bacteria. In the human gut microbiota, bacteria are by far the most abundant microorganisms and are composed of many species that can be taxonomically classified by their genus, family, order, and phylum. The main microbial phyla dominating the GI tract of healthy adults are *Firmicutes* and *Bacteroidetes*, followed by *Actinobacteria*, *Proteobacteria*, *Fusobacteria*, and *Verrucomicrobia*. Within this microbial ecosystem, the majority of bacterial species inhabiting the human adult gut typically belong to two phyla: the Firmicutes (*Clostridium*, *Enterococcus*, *Lactobacillus*, and *Ruminococcus*) and the Bacteroidetes (which includes *Bacteroides* and *Prevotella* as predominant genera) [33,34] (Figure 1).The microbial density and composition vary across the GI tract, with increasing bacterial abundance and phylogenetic diversity observed from the proximal to the distal GI tract regions [35]. The human endogenous intestinal microbiota includes approximately >4000 bacterial species, comprising a bulk of genetic material that is estimated to be 150 times greater than the human host genome [32,36]. The gut microbiota maintains a constant dynamic and homeostatic state in the host. In mammals, gut bacteria are categorized into two primary functional groups: commensal symbionts, which are beneficial and support host health by maintaining homeostasis, and pathobionts, which are resident microbes that remain harmless under normal conditions but can become pathogenic if their abundance increases. Under normal physiological conditions, commensal symbionts predominate and maintain a symbiotic relationship with the host. However, a disruption of this balance can lead to pathobiont overgrowth, triggering an inflammatory response that exerts harmful effects on the host [37]. GI tract bacteria are key regulators of digestion; they play a crucial role in the extraction, synthesis, and absorption of many nutrients and metabolites, including bile acids, amino acids, vitamins, and short-chain fatty acids (SCFAs). The gut microbiota has an important immune function against pathogenic bacteria colonization using many processes such as nutrient metabolism, pH modification, and antimicrobial peptide secretions that help to maintain intestinal epithelium integrity [38]. Recently, due to the development of high-throughput omics technologies, such as next-generation sequencing platforms [39], our knowledge of the microbiota in relation to intestinal and extra-intestinal diseases has increased considerably [40], enabling us to identify potential role of the gut microbiota composition and gut microbiota-derived metabolites in autoimmune and metabolic disorders in humans [41], including GDM [42]. Therefore, to maintain the optimal immune and metabolic functions, an optimal gut microbiota composition remains fundamental, particularly during pregnancy.

## 3. Gut Dysbiosis During Pregnancy and Its Link to GDM

During pregnancy, women’s bodies undergo substantial hormonal, metabolic and immunological changes to support maternal physiology demands and ensure optimal fetal growth and development [43]. During the first trimester of pregnancy, there is an increase in maternal insulin secretion, which enhances glucose uptake and lipogenesis, facilitating energy storage in preparation for the elevated metabolic demands for healthy fetal development, which subsequently leads to weight gain in pregnant women. As the pregnancy progresses, the levels of pro-inflammatory cytokines and metabolic hormones such as human placenta lactogen (hPL), estrogen and luteinizing hormone increase, which stimulates additional insulin secretion [44]. In pregnant women most susceptible to GDM, insulin production, which inadequately compensates for the insulin-inhibiting effect proposed to be driven by the high levels of placental hormones (e.g., estrogen, and hPL) during mid-gestation, is impaired due to pancreatic β-cell dysfunction [6,45], thereby contributing to GDM [46]. Increased maternal age, obesity, and a family history of any form of diabetes are well-known risk factors for the development of GDM. In addition, recent clinical evidence has linked gut dysbiosis, which is defined as an imbalance of gut microbiota composition, with GDM [30]. Moreover, a growing number of longitudinal and cross-sectional human case–control studies, as well as animal experiments, have implicated altered gut microbiota composition in the pathogenesis of GDM [29]. Pregnant women with GDM often exhibit lower bacterial richness (called intra-individual richness or α-diversity) with a decreased abundance of a group of bacteria belonging to commensal symbionts such as *Romboutsia*, *Lactobacillus*, *Actinomyces*, *Verrucomicrobium*, *Ruminococus*, *Akkermansia*, *Escherichia-Shigella*, *Bifidobacterium*, *Clostridium*, *Rothia*, and *Corynebacterium* [47,48]. This finding has been evidenced in several human trials. For example, Zheng et al. [49] reported a decreased abundance of *Coprococcus* and *Streptococcus* in women with GDM compared to healthy, normoglycemic pregnant women. Moreover, in a study conducted in Chengdu, China, Hu et al. [47] reported a significant decrease in *Actinobacteria*, and *Bifidobacterium* in women with GDM. Furthermore, they demonstrated that the reduction in the abundance of *Clostridium* and *Corynebacterium* was positively correlated with fasting blood glucose, as well as 1 h and 2 h postprandial blood glucose levels. In contrast, the abundance of *Akkermansia* was negatively correlated with 1 h postprandial blood glucose level and positively associated with insulin sensitivity [50]. In support of these findings, Sun and al. found that an abundance of Bacteroides massiliensis, a Gram-negative anaerobic bacteria, was associated with GDM, and *Mycobacterium* and *Anaerostipes hadrus* were associated with glucose intolerance [51]. Notably, adding data from the gut microbiota into the models predicting individual postprandial glycemic response (PPGR) showed that gut microbial features are some of the most impactful individual parameters on PPGR prediction in pregnant women with GDM [52]. Additionally, Pinto et al. demonstrated that gut dysbiosis linked to GDM was proposed to be driven by dysbiosis-induced inflammation months before clinical diagnosis [23]. This link was evidenced by elevated pro-inflammatory cytokines, such as IL-6, IL-8, and TNF-α, during the first trimester and reduced levels of fecal branched SCFAs, such as isovalerate and isobutryrate, which play a role in suppressing inflammatory responses. The involvement of the intestinal microbiota in GDM was further evidenced by fecal microbiota transplantation (FMT) from GDM to germ-free (GF) mice [53,54]. Stool transplantation samples from donors with GDM into GF mice led to hyperglycemia, indicating an effect of the gut microbiota on glucose metabolism [54]. Similar results were found by Frishman S. et al. [23] showing that fecal microbiota transplant from women in their first trimester of pregnancy led to impaired glucose tolerance in germ-free mice, which was reversed after receiving transplant from the same women following dietary intervention treatment. The findings of these studies, despite being associative-based evidence in humans (Table 1), highlight the role of the gut microbiota in the pathogenesis of GDM. However, significant limitations remain due to heterogeneity across studies, including differences in cohort characteristics (e.g., sample size, GDM diagnostic criteria, ethnicity, lifestyle, and dietary patterns), study design, timing of sample collection during pregnancy, and non-standardized gut microbiota sequencing methods (16S rRNA versus shotgun metagenomics). Additionally, how gut dysbiosis leads to the development of GDM is not fully understood. Recently, various studies have proposed several potential mechanisms by which gut dysbiosis may contribute to glucose intolerance and, subsequently, diabetes and/or GDM. These include alterations in metabolic–immune responses and a disruption of gut barrier integrity.

## 4. Intestinal Microbiota-Derived SCFAs in GDM Pathogeny

**Figure 2:**
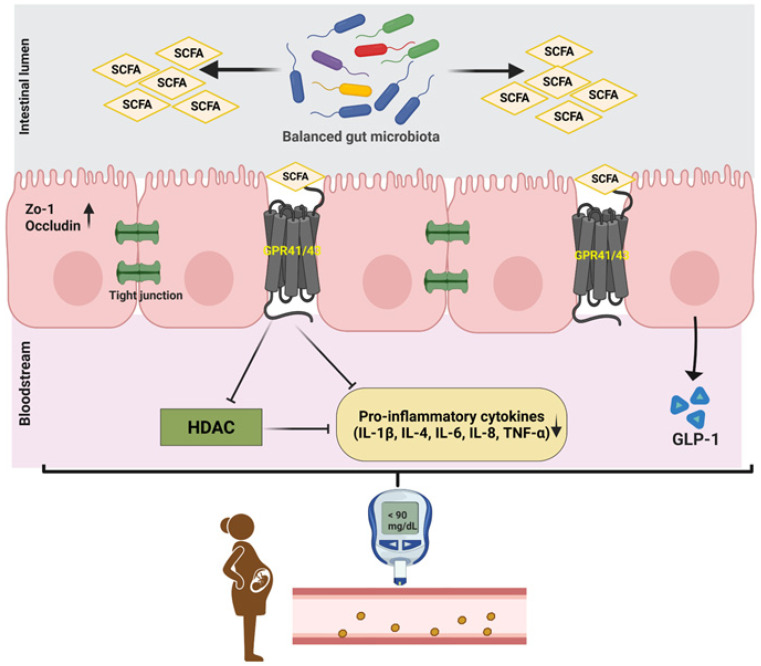
Mechanisms of gut microbiota-derived SCFAs on glucose homeostasis in women with GDM. Normal or balanced gut microbiota might have sufficient SCFA-producing bacteria. Elevated activity of SCFA-producing microbiota improves dietary fermentation and promotes synthesis and secretion of SCFAs such as butyrate, acetate and propionate. SCFAs have potential in inhibiting pro-inflammatory cytokines (e.g., IL-1β, IL-4, IL-6, IL-8, TNF-α) through activation of GPR41/43. The anti-inflammatory properties of SCFAs are mediated through the inhibition of histone deacetylase (HDAC) and/or upregulation of tight junction proteins (occludin, Zo-1). Moreover, SCFAs stimulate GLP-1 secretion. SCFAs: short chain fatty acids; Zo-1: zonula occludens-1; GPR41/43: G protein-coupled receptor 41/43. GLP-1: glucagon-like peptide-1; HDAC: histone deacetylase; IL-1β: Interleukin-1 beta; IL-4: Interleukin-4; IL-6: Interleukin-6; IL-8: Interleukin-8; TNF-α: tumor necrosis factor-alpha. Created in BioRender. Al-Siddiqi, H. (2026) https://BioRender.com/dn79wp9 (accessed on 16 April 2026).

Due to the lack of specific enzymes required to digest dietary fibers, the human GI tract relies on the gut microbiota to synthetize and produce short-chain fatty acids (SCFAs) [55]. SCFAs such as butyrate, acetate and propionate, which constitute over 95% of total SCFA contents, are intestinal microbial-derived metabolites produced in the large intestine through the fermentation of “indigestible” dietary prebiotics. SCFAs are absorbed in the intestine, where acetate is the most predominant SCFA by concentration. However, butyrate serves as the primary energy source for colonic epithelial cells, providing approximately 60–70% of their total energy requirement through mitochondrial β-oxidation. This process promotes intestinal epithelial cell growth and enhances gut barrier integrity, thereby preventing abnormal intestinal permeability, a phenomenon commonly referred to as “leaky gut”. SCFAs function as key signaling molecules between the gut microbiota and the host, acting both locally within the intestine and systemically via the portal vein, and are believed to mediate the health-promoting effects associated with fiber-based diets. SCFAs are known to promote glucose homeostasis and have also been demonstrated to inhibit the production of pro-inflammatory mediators, including TNF-α, IL-1β, and IL-6 [56]. Under healthy conditions with a balanced gut microbiota, a sufficient intake of dietary fiber promotes the expansion of SCFA-producing bacteria. This increases both the local fecal concentrations and systemic levels of SCFAs, which in turn improves glucose homeostasis [57,58,59]. Conversely, dysbiotic gut, which is reported in cases of GDM, has been linked to reduced SCFA production, a factor that may potentially contribute to insulin resistance and inflammation during pregnancy. Importantly, large prospective cohort studies have shown that high dietary fiber intake during pregnancy significantly improved glucose metabolism and reduced inflammation by enhancing the activity of SCFA-producing gut microbial genera through fermentation [60,61]. However, the underlying molecular mechanisms are not fully understood. Data from animal studies suggested potential pathways linking dysbiosis to pregnancy-induced insulin resistance; one prominent mechanism involves the alteration of the gut metabolite-sensing receptors, specifically free fatty acid receptor 2 (FFAR2, also known as GPR43) and FFAR3 (GPR41) within metabolically active tissues, including pancreatic islets [62]. Moreover, microbial-derived SCFAs act as a potent endogenous agonist for FFAR2 [63], suggesting a critical link between the gut microbiota, SCFAs, and FFAR2 during pregnancy. FFAR2, a deorphanized G protein-coupled receptor (GPCR) known to exert many metabolic functions, is highly expressed in pancreatic islets during pregnancy. FFAR2 activation results in increased insulin secretion, essential for adequate gestational glucose homeostasis. Studies have shown that pregnant mice lacking *Ffar2* exhibited impaired glucose tolerance, reduced β-cell expansion and lower SCFA levels. These findings suggest that the impaired production of gut microbiota-derived metabolites (e.g., SCFAs) resulted from an altered gut microbiota, which may contribute to GDM through an *Ffar2*-dependent signaling pathway [62]. Additionally, SCFAs have been shown to activate enteroendocrine L cells and stimulate incretin secretion, specifically glucagon-like peptide-1 (GLP-1), in both human and rodents [64,65,66]. GLP-1 promotes insulin secretion and regulates glucose metabolism by promoting glycogen storage and suppressing glycolysis in the liver and muscles [67,68]. In primary murine colonic cultures, SCFAs significantly stimulated GLP-1 secretion via *Ffar2*-dependent signaling pathway, as knockout of *Ffar2* impaired GLP-1 secretion in response to acetate and propionate stimulation [69,70]. These data demonstrated that SCFA-induced GLP-1 secretion constitutes another underlying mechanism by which the gut microbiota may contribute to glucose homeostasis. Studies showed that elevated levels of pro-inflammatory cytokines (e.g., IL-4, IL-6, IL-8, TNF-α) found in pregnant women diagnosed with GDM are associated with lower levels of SCFAs [23], indicating that SCFAs have a potential role in reducing the inflammatory response [71]. Their immunomodulatory effect has been demonstrated in co-culture models showing that butyrate administration significantly suppressed the inflammatory activity generated by the interaction of adipocytes and macrophages through reduced lipolysis via activation of the G protein-coupled receptor GPR41 pathway and the inhibition of pro-inflammatory cytokines, such as TNF-α, MCP-1, and IL-6 [71]. In addition to the activation of GPR41, the anti-inflammatory properties of SCFAs are mediated through the inhibition of histone deacetylase (HDAC) [56]. SCFAs are also known to reduce low-grade inflammation through the upregulation of tight junction proteins (occludin, zonula occludens-1 (Zo-1)), thus promoting intestinal barrier function (e.g., reduced gut permeability) [72], suggesting potential for SCFAs as therapeutic factors against dysbiosis-induced metabolic endotoxemia (Figure 2).

## 5. Gut Microbiota-Derived LPS in GDM Pathogeny

Insulin resistance is associated with elevated inflammatory markers; numerous studies have reported that women with GDM exhibited increased plasma levels of inflammatory cytokines, which are linked to reduced SCFAs and elevated Lipopolysaccharide (LPS) levels [23,73,74,75,76]. LPS is a major component of the outer membrane of Gram-negative bacteria cell walls and shares a conserved structural architecture. LPS is a large molecule composed of three parts: an outer core polysaccharide, an inner core oligosaccharide and Lipid A. LPS induces innate immunity and triggers acute inflammatory responses by stimulating the release of pro-inflammatory cytokines, such as IL-1β, IL-6, and TNFα, in a variety of cell types [77], particularly monocytes and macrophages [78]. Consequently, gut dysbiosis increases LPS availability, leading to low-grade inflammation. These altered cytokine profiles suggest that gut dysbiosis-induced inflammation during pregnancy may contribute to the pathogenesis and progression of GDM.

**Figure 3:**
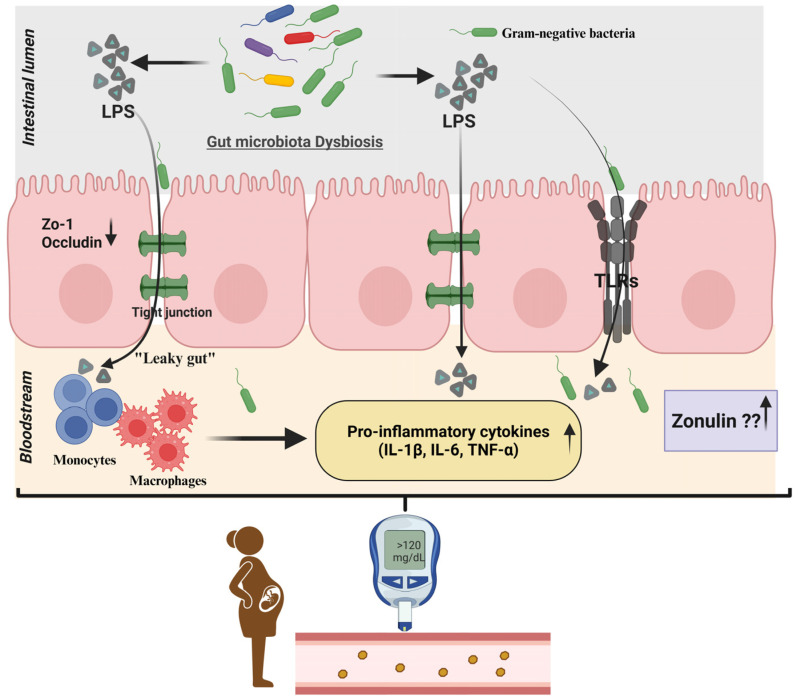
Proposed mechanisms of gut microbiota-derived LPS in the pathophysiology of GDM. Gut microbiota dysbiosis leads to elevated Gram-negative bacteria. Increased abundance of Gram-negative bacteria triggers LPS overproduction. The expansion of Gram-negative pathobionts increases gut permeability—a phenomenon referred to as “leaky gut”—through the downregulation of tight junction proteins (e.g., occludin, Zo-1), thus facilitating LPS translocation into the bloodstream through TLRs. Microbiota-derived LPS has the potential to activate monocytes and macrophages, leading to the upregulation of pro-inflammatory cytokines (e.g., IL-1β, IL-6, IL-8, TNF-α). Increased plasma level of zonulin, a physological modulator of tight junctions, could be a potential biomarker of a “leaky gut”. LPS: Lipopolysaccharide; Zo-1: zonula occludens-1; TLRs: toll-like receptors; IL-1β: Interleukin-1 beta; IL-6: Interleukin-6; TNF-α: tumor necrosis factor-alpha; ?? means potential biomarker. Created in BioRender. Al-Siddiqi, H. (2026) https://BioRender.com/9tyucjf (accessed on 16 April 2026).

Mucosal layer and tight junction proteins, such as occludin and ZO-1, within the gut epithelial layer play crucial roles in defending the host against invasive pathobionts. In animal models of diabetes, gut microbiota dysbiosis has been associated with impaired intestinal epithelial barrier integrity through the downregulation of tight junction protein expression [79,80], resulting in a condition commonly referred to as “leaky gut”. Recent studies have shown that plasma zonulin, a physiological modulator of tight junctions and a predictor of gut permeability, is significantly upregulated in women with GDM [81,82,83]. Zonulin has also been suggested to be associated with insulin resistance and may thereby contribute to the development of GDM. A single prospective longitudinal study of 314 pregnant women at the Volta regional hospital, Ghana, reported a markedly elevated odds ratio for GDM among pregnant women with high first-trimester plasma zonulin. In their regression analysis models 1 and 2, one unit (1 ng/mL) increase in the zonulin level increased the odds of GDM by 1.25 and 1.26, respectively [84]. Moreover, a pilot study comprising 88 women from southwest Finland reported that early pregnancy serum zonulin concentration was associated with higher odds of GDM at mid-pregnancy (OR for 1 unit increase in zonulin: 1.08, 95% CI: 1.02–1.15; *p* = 0.009) [81], suggesting that the early pregnancy circulating zonulin level could be used as a potential predictor for GDM. However, future studies in large and multiethnic cohorts are required before endorsing zonulin as a reliable clinical biomarker for GDM. Additionally, women with GDM exhibit an increased proportion of Gram-negative bacteria associated with the upregulation of pathways related to LPS biosynthesis [2,22]. This rise in Gram-negative pathobionts in women with GDM, initiated by gut dysbiosis, may lead to increased gut permeability, facilitating LPS translocation from the gut lumen into the lymphatic system and subsequently into the bloodstream. This phenomenon primarily triggers inflammatory pathways and low-grade inflammation by binding to and activating toll-like receptors, subsequently leading to glucose intolerance. These data highlight that LPS-induced low-grade inflammation is a key mechanism through which gut dysbiosis may contribute to the pathogeny of GDM (Figure 3). Overall, gut microbiota may contribute to the metabolic changes associated with GDM through the mechanisms described above. However, further studies are still needed to fully understand the molecular pathways involved in gut microbiota-induced GDM.

## 6. Gut Microbiota in GDM: Multi-Omics Integration to Early Prediction and Precision Medicine

Given the complex pathophysiology of GDM, along with the variability in patient responses to lifestyle-based interventions (e.g., diet and physical activity) and the adverse effects commonly associated with pharmacological treatments [85,86,87], there is a growing need for a personalized care model to manage GDM. The Precision Medicine in Diabetes Initiative, launched in 2018 by the American Diabetes Association in collaboration with the European Association for the Study of Diabetes [85], aims to utilize patient-specific biomolecular data to tailor diagnostic, preventive, and therapeutic strategies in a timely and cost-effective manner [88,89]. With the emergence of high-throughput OMICS technologies, substantial efforts have been directed toward integrating comprehensive data from fundamental biomarkers (i.e., genes, RNA, proteins, and metabolites) to achieve a holistic understanding of the human disease, thereby enhancing diagnosis and therapeutic strategies [90,91]. More importantly, advances in next-generation sequencing technologies [92], including 16S rRNA gene sequencing and shotgun whole-genome sequencing—two principal approaches used in gene expression profiling—have substantially enhanced our understanding of the composition and functional dynamics of the gut microbiota. In contrast to 16S rRNA-based sequencing, the shotgun metagenomic approach comprehensively sequences the entire genomic content of a sample, including regions beyond the conserved 16S rRNA locus. This enables higher-resolution taxonomic profiling, facilitating accurate identification at both species and strain levels [93]. However, its elevated cost and the need for more complex bioinformatics analyses constrain its broader implementation and routine utilization. Thus, for stool samples, shotgun sequencing remains recommended, while 16S rRNA is more suitable for tissue samples [93]. For multifactorial metabolic diseases such as diabetes, integrating high-throughput next-generation sequencing data (genomics, epigenomics and transcriptomics) with mass spectrometry-based OMICS (proteomics, metabolomics) is essential to unravel the complex interactions between genes, environmental factors, and molecular pathways involved in disease development, ultimately enabling more effective therapeutic approaches. In light of these insights, and given the highly personalized nature of gut microbiota composition and the hormonal shifts during the first and third trimesters of pregnancy that alter microbial communities [94], integrating microbiomics with diverse multi-omics platforms is essential for a comprehensive understanding of the host–microbe interactions associated with the development of GDM. This integration may enhance early prediction and diagnosis, enabling early intervention and personalized treatment strategies for the management of GDM. In a metabolome-wide association study, Susarla et al. [95] demonstrated that microbiota-derived metabolites, the end-products of gut microbial metabolism, can serve as early biomarkers of GDM. They found that branched-chain amino acids (e.g., leucine, isoleucine, valine) at 10–13 weeks of gestation and unsaturated fatty acids at 16–19 weeks of gestation were positively associated with risk of developing GDM. Furthermore, Guan et al. analyzed the gut microbiota composition of infertile women undergoing frozen embryo transfer to identify pre-pregnancy biomarkers for GDM, with samples collected at three different time points: pre-pregnancy, first trimester (T1), and second trimester (T2). They found that women who later developed GDM had a lower abundance of beneficial butyrate-producing bacteria prior to pregnancy, while a higher abundance of *Bacteroides dorei* occurred during the second trimester, suggesting a potential link between this species and the development of GDM [96]. At the pre-pregnancy period, the difference in the gut microbiota was proposed to be driven by specific taxa (with *Prevotella copri* being the most prominent pre-pregnancy difference) rather than changes in the overall microbiota community. The Prevotella strain belongs to a mucin oligosaccharide chain-degrading bacteria involved in the thinning of the intestinal epithelial layer [97], a mechanism that triggers “leaky gut”, thereby contributing to the pathogenesis of GDM. These findings indicate that gut microbiota differences between women who have a higher risk of developing GDM exist before pregnancy. Collectively, these findings demonstrated that gut dysbiosis during pre- and/or early stages of pregnancy can progress to GDM and thus can be used as an early prediction tool, providing a valuable window for early prediction and personalized dietary intervention for prevention. However, large multiethnic studies are needed to validate the utility and applicability of this microbiota-based biomarker approach.

The importance of integrating gut microbiota data with diverse OMICs platforms to enhance prediction tools for the early detection of GDM has been demonstrated in several studies. A recent study by Pinto Yishay et al. [23] identified a significant reduction in branched short-chain fatty acids (BSCFAs), particularly isovalerate and isobutyrate, in women who later developed GDM. Furthermore, they demonstrated that integrating first-trimester microbiota-derived metabolite signatures with conventional clinical data (e.g., BMI and age), dietary habits, and inflammatory cytokine profiles increased the predictive accuracy—the model’s ability to correctly classify at-risk individuals—from an AUROC of 0.73 to 0.83. In another study by Ma et al. [98], conducted on a cohort of 98 matched Chinese pairs of pregnant women (10–15 weeks of gestation), the authors demonstrated that their Linear Discriminant Analysis (LDA) prediction model, which combined five bacterial genera with clinical data (e.g., fasting blood glucose), achieved an AUROC of 0.736 (in the discovery set) and 0.696 (in the validation set). This integrated prediction model outperformed both its individual components (microbiota or clinical data alone) and established external clinical risk-prediction models, which showed lower performance when validated against their independent cohort. Additionally, they identified that starch and sucrose metabolism pathways were positively correlated with fasting blood glucose (r = 0.194), while the lysine biosynthesis pathway was negatively associated (r = −0.181), suggesting that gut dysbiosis may actively contribute to the development of GDM. However, due to the small cohort size and geographical influence on gut microbiome composition and diversity, more large-scale multiethnic/multi-location cohort studies are required to ascertain this finding. Collectively, these studies highlight the importance of integrating multi-omics data to enhance the performance of AI models designed to identify individuals at risk of GDM before clinical diagnosis. However, most of the currently reported AI models in GDM prediction remain exploratory and are not generalized across diverse populations. Also, overfitting remains a major limitation of AI-based gut microbiota prediction models due to the high dimensionality of the gut microbiota data relative to typically small cohort sizes commonly reported in the literature. To address the risk of overfitting, larger, adequately powered, and externally validated datasets are therefore required to ensure the robustness of AI models prior to clinical implementation.

## 7. Intervention to Reverse or Slow Down the Progression of GDM

Dietary intervention through balanced, low-glycemic-index diets is the first-line management option for GDM. A systematic review and network meta-analysis involving a total of 28 randomized controlled trials with 2666 participants evaluating distinct dietary interventions either alone or in combination (low-glycemic-index diet (Low-GI), Dietary Approaches to Stop Hypertension (DASH) diet, low-glycemic-load diet, low-carbohydrate diet, and standard diet) reported that the DASH diet was the most effective intervention for glycemic control, while both the DASH and Low-GI diets significantly reduced adverse pregnancy outcomes [99]. Although the authors suggest that DASH and Low-GI diets may be beneficial for managing GDM, further multiethnic and multiregional well-designed studies using large cohorts are still required to validate these findings and support evidence-based clinical recommendations. Diet is the primary driver of gut microbiota composition. Therefore, the gut microbiota holds great promise for transforming personalized care in GDM, particularly personalized dietary interventions based on machine learning algorithms that integrate gut microbiota data, dietary habits, and clinical data (e.g., blood biochemical parameters, BMI, and age) to successfully and accurately predict the impact of diet on a patient’s postprandial glycemic response [100]. In a randomized crossover dietary intervention trial, Korem et al. have demonstrated that, although the individual gut microbiota profile is personalized and can be resistant to dietary intervention, by comparing gut microbiota changes among individuals consuming white versus sourdough bread, the gut microbiota signature is unique to each individual and can be used to predict glycemic response to dietary habits [101]. Several randomized controlled trials studied the effect of probiotics on the health of both mothers and infants. In a double-blind study, Lactobacillus rhamnosus (HN001), a well-studied probiotic, was found to have beneficial effects on reducing the prevalence of GDM [102]. In this New Zealand double-blind, randomized placebo-controlled study [102], an HN001 supplement was given to a group of women from early pregnancy to delivery or 6 months postpartum (for the breast-feeding group). The HN001 supplement reduced the prevalence of GDM from 13.8% among women who did not receive it to 8.2% among those who did. This effect was more pronounced among older women and those with a history of GDM; thus, HN001 is believed to have a protective effect in high-risk groups. HN001 alters the gut microbiota, and thus can improve insulin sensitivity and reduce inflammation. This study highlights a potential targeted intervention for women at high risk of developing GDM. However, due to the lack of pre-pregnancy maternal anthropometric measures and relatively smaller sample size, more research is needed to confirm these findings. In another study [103], the daily intake of synbiotic capsules containing Lactobacillus acidophilus, Lactobacillus casei and Bifidobacterium bifidum (2 × 109 CFU/g each) plus 0.8 g inulin for 6 weeks in pregnant women with GDM in their late second and early third trimester led to reduced fasting serum insulin and insulin resistance (HOMA-IR) when compared to a placebo group. Using metagenomic sequencing, a more accurate approach than 16S rRNA sequencing, on stool samples from overweight or obese pregnant women with and without GDM in southwest Finland, Mokkala et al. reported that only women without GDM exhibited significant alterations in bacterial species abundance during pregnancy in response to dietary supplementation combining fish oil and probiotics, suggesting that GDM status may prevent maternal gut microbiota flexibility, thus limiting the capacity of women with GDM to respond to prebiotics and probiotics [104]. In addition to clinical findings, pre-clinical studies reported a positive effect of probiotic supplementation on fasting blood glucose levels in GDM rat models. Using 16S rRNA sequencing, Zheng et al. found a decreased diversity in the gut microbiota of the GDM group when compared with the non-GDM control group. This reduction in gut microbiota diversity was restored after probiotic supplementation. In particular, the abundance of *Firmicutes* and *Actinobacteria* increased after probiotic supplementation [105]. Controversially, a recent prospective double-blind randomized controlled trial of probiotic versus placebo, given from the second trimester in overweight and obese pregnant women, revealed no effect of the probiotics on the incidence of GDM at 28 weeks of gestation [106]. This is consistent with evidence from seven trials with 1647 participants reporting no clear effect of probiotics on the risk of GDM [107]. Additionally, the authors showed an increased risk of pre-eclampsia, a pregnancy-related complication, with probiotic supplementation [107]. Similarly, an umbrella meta-analysis of 27 studies involving 83,817 participants confirmed an increased risk of pre-eclampsia associated with probiotic supplementation during pregnancy (RR = 1.23, 95% CI: 1.07–1.42) [108]. In contrast, a recent systematic review and meta-analysis of 29 trials involving 7735 pregnant women found no significant association between probiotic supplementation and pre-eclampsia risk (RR: 1.14, 95% CI: 0.84–1.53) [109]. However, substantial heterogeneity in participant populations, the type of probiotic tested (>20 different strains) and intervention protocols across the included trials may explain this discrepancy, suggesting that further high-quality trials are needed to definitively assess the benefits and possible harms of probiotic supplementation during pregnancy.

With gut microbiota dysbiosis emerging as a potential contributing factor to the pathogenesis of diabetes mellitus, restoring its homeostasis through fecal microbiota transplantation (FMT) may represent a new therapeutic strategy for T2DM and GDM. While FMT has been demonstrated to favorably modulate glucose and insulin metabolism in T2DM animal models by restoring gut microbiota balance [20,110,111], studies on its therapeutic potential and safety in GDM are still lacking. Therefore, further preclinical studies are required to explore the efficacy and safety of FMT in GDM. Besides FMT, several pharmacological drugs are widely used to treat GDM. Recently, it was demonstrated that the gut microbiota can directly influence an individual’s response to a specific drug by enzymatically transforming its structure and subsequently altering its bioactivity, bioavailability or toxicity (a term known as pharmacomicrobiomics) [112]. This applies more specifically to orally administered drugs that transit through the GI tract where they interact with different species of bacteria residing in the gut. Conversely, the composition and function of the gut microbiota can be modulated by pharmacological drugs, including non-antibiotic drugs, suggesting a bidirectional interaction between drugs and the gut microbiota [113,114]. Metformin, the first-line antidiabetic drug commonly prescribed to pregnant women with GDM, appears to exert some of its therapeutic effect by modulating the gut microbiome [115]. This beneficial effect is mediated primarily through an increase in microbiota-derived SCFAs [114]. Additionally, acarbose, an alpha-glucosidase inhibitor, is recommended in many countries as a safe alternative to metformin for glycemic control in T2DM [116]. Acarbose primarily exerts its antidiabetic effects by inhibiting the hydrolysis of complex carbohydrates in the small intestine, thereby reducing glucose absorption. In a prospective, open-label, controlled study to evaluate maternal–fetal outcomes in patients with GDM treated with insulin versus acarbose until six weeks after delivery, Jayasingh Sr et al. reported similar neonatal and maternal outcomes and glycemic control (fasting and postprandial blood glucose levels) between the two groups [117], suggesting acarbose as an effective and promising therapy option for GDM. Moreover, a network meta-analysis, including 32 RCTs to compare and rank different antidiabetic drugs for glucose control and pregnancy outcomes in GDM patients, ranked acarbose as the best in reducing the risk of neonatal hypoglycemia, followed by metformin [118]. Within the GI tract, acarbose exposure leads to an increased concentration of complex carbohydrates, which potentially affects the gut microbiota composition by increasing the relative abundance of Lactobacillus and Bifidobacterium and reducing Bacteroides species, including Bacteroides thetaiotaomicron, thereby raising the relative abundance of bacteria involved in bile acid metabolism [119]. This suggests that the beneficial effects of acarbose on GDM may be attributed to its favorable role in promoting a healthy gut microbiota.

**Figure 4:**
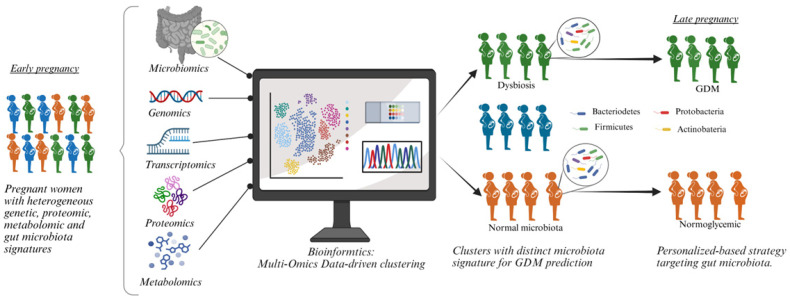
Overview of incorporating microbiomics into other omics (genomics, transcriptomics, proteomics, metabolomics) that can help to drive the development of microbiota-based precision medicine in GDM. Illustration of multi-omics data integration approach highlighting gut microbiota in personalized medicine, including diagnosis, prognosis, prevention and management of GDM. GDM, gestational diabetes mellitus. Created in BioRender. Al-Siddiqi, H. (2026) https://BioRender.com/3octvp2 (accessed on 16 April 2026).

Overall, although substantial improvements of metabolic parameters following probiotic and prebiotic administration were reported, their impact on GDM incidence and progression and the reported potential adverse effects, such as an increased risk of pre-eclampsia, highlight the need for further investigation to better understand the efficacy and safety of probiotic and prebiotic supplements during pregnancy. Additionally, the discrepancies in findings among studies may be related to some confounding factors such as pre-pregnancy body mass index, weight gain, maternal dietary choices and antibiotic use during the gestational period, all known to impact the gut microbiota composition of pregnant mothers, thereby highlighting the importance of personalized approaches for prebiotic- and probiotic-based interventions in managing GDM. Also, variations in experimental design (e.g., sample inclusion and exclusion criteria, small cohort size) in these studies affected the strength and accuracy of the developed prediction tools and made it difficult to use microbiota alone for the prediction of the development of GDM. Therefore, we recommend multi-omics analysis integration to validate data/findings using different populations. Together, these studies highlight the importance of integrating microbiomics (person-specific factors) into the current treatment guidelines for GDM management. This further supports the application of precision medicine approaches to achieve more effective, optimal and rapid individualized care that benefits both mothers and their offspring (Figure 4).

## 8. Conclusions

GDM is a multifactorial metabolic disorder, characterized by abnormal glucose metabolism, that primarily occurs in the mid to late stages of pregnancy. Hyperglycemia during pregnancy has been shown to have short- and long-term health risks to both the mother and the fetus. Over the past years, studies, despite being based on associative evidence in humans, have reported a link between altered gut microbial composition and reduced microbial diversity, termed as “dysbiosis”, and the pathogenesis of GDM. This article provides a comprehensive overview of recent studies, highlighting the association between gut microbiota dysbiosis and GDM. The underlying mechanisms discussed in this review were related to the impact of gut dysbiosis on microbiota-derived SCFAs and LPS in the pathogenesis of GDM. Moreover, based on its modifiable and person-specific profile nature, unlike genetics, the gut microbiota holds considerable potential as a tool for precision medicine. Thus, this review also emphasizes the importance of integrating microbiomics with other OMICs data from fields such as genomics, transcriptomics, proteomics, and metabolomics, which hold promise for advancing the diagnosis, prevention and treatment of GDM. The integration of these multi-omics, including microbiomics data, into machine learning models could pave the way for early diagnosis and personalized management of GDM, ultimately improving maternal–infant health outcomes. However, regarding clinical translation, key limitations remain, such as the heterogeneity of existing findings due to study cohort characteristics (small size, GDM diagnostic criteria, ethnicity, lifestyle, dietary habits), sampling time (first, second, third trimesters of pregnancy) and a lack of standardized methodologies in gut microbiota sequencing (16S rRNA versus shotgun sequencing). While findings are promising, the following key research questions must be addressed before clinical implementation: 1. How can GDM diagnostic criteria and microbiota sequencing-based methods be standardized? 2. Which specific gut microbiota taxa and microbiota-derived metabolites derive from GDM and serve as universal diagnostic biomarkers? 3. What factors determine individual responses to microbiota-targeted intervention for GDM? 4. How can the integration of microbiomics with other omics (genomics, metabolomics, proteomics) improve the validation of predictive biomarkers for GDM across diverse populations?

## Figures and Tables

**Table 1:** Association between gut microbiota and GDM. T1: the first trimester; T2: the second trimester; T3: the third trimester; GDM: gestational diabetes mellitus.

Year	Participants	Sampling Time	Sequencing Methods	GDM Associated Gut Microbiota		References
				Increased	Decreased	
2025	77 GDM 28 Healthy	≥24 weeks	16S rRNA sequencing	Gut microbiome features rank among the top parameters influencing the postprandial glucose responses prediction in women with GDM		[52]
2024	6 GDM 7 Healthy	T1, T2, T3	16S rRNA sequencing	Proteobacteria	Firmicutes	[24]
2023	44 GDM 350 Healthy	T1	16S rRNA sequencing	Inflammatory cytokines (IL-6)	*Prevotella copri* SCFAs	[23]
2023	50 GDM 54 Healthy	24–28 weeks	Shotgun sequencing	Verrucomicrobia *Bacteroides eggerthii*, Megamonas unclassified *Ruminococcus gnavus*	Faecalibacterium, Prevotella, Streptococcus *Prevotella copri, Prevotella stercorea* Prevotella/Bacteroides ratio	[42]
2021	147 GDM 271 Healthy	25–26 weeks	16S rRNA sequencing	Firmicutes	Proteobacteria	[21]
2021	201 GDM 201 Healthy	6–15 weeks	16S rRNA sequencing	Enterobacteriaceae, *Ruminococcaceae* spp., and Veillonellaceae	Rothia, Actinomyces, Bifidobacterium, Adlercreutzia, Coriobacteriaceae and *Lachnospiraceae* spp.	[47]
2021	21 GDM 32 Healthy	24–28 weeks	16S rRNA sequencing	Bacteroidetes Citrobacter Parabacteroides Fusicatenibacter	Proteobacteria Actinobacteria Verrucomicrobia	[50]
2020	31 GDM 103 Healthy	T1, T2	16S rRNA sequencing	Holdemania, Megasphaera, and Eggerthella	Coprococcus and Streptococcus	[49]
2020	45 GDM 45 Healthy	T1, T2	16S rRNA sequencing	Blautia and Faecalibacterium	Akkermansia, Odoribacter, Butyricimonas, and Christensenellaceae	[54]
2018	41 GDM	24–28 weeks and 38 weeks	16S rRNA sequencing	Firmicutes	Bacteroidetes and Actinobacteria	[22]
2018	50 GDM 157 Healthy	T3	16S rRNA sequencing	Faecalibacterium Anaerotruncus	Clostridium Veillonella	[30]
2017	43 GDM 81 Healthy	21–29 weeks	Shotgun sequencing	*Parabacteroides distasonis, Klebsiella variicola*	*Methanobrevibacter smithii*, *Alistipes* spp. *Bifidobacterium* spp. *Eubacterium* spp.	[2]

## Data Availability

No new data were created or analyzed in this study. Data sharing is not applicable to this article.
